# An effective biomedical data migration tool from resource description framework to JSON

**DOI:** 10.1093/database/baz088

**Published:** 2019-07-25

**Authors:** Jian Liu, Mo Yang, Lei Zhang, Weijun Zhou

**Affiliations:** 1 School of Computer Science and Technology, Harbin Institute of Technology, Harbin, China; 2 Zhejiang University of Science and Technology, Hangzhou, China; 3 Department of Hematology, Zhujiang Hospital, Southern Medical University, Guangzhou, China

## Abstract

Resource Description Framework (RDF) is widely used for representing biomedical data in practical applications. With the increases of RDF-based applications, there is an emerging requirement of novel architectures to provide effective supports for the future RDF data explosion. Inspired by the success of the new designs in National Center for Biotechnology Information dbSNP (The Single Nucleotide Polymorphism Database) for managing the increasing data volumes using JSON (JavaScript Object Notation), in this paper we present an effective mapping tool that allows data migrations from RDF to JSON for supporting future massive data explosions and releases. We firstly introduce a set of mapping rules, which transform an RDF format into the JSON format, and then present the corresponding transformation algorithm. On this basis, we develop an effective and user-friendly tool called RDF2JSON, which enables automating the process of RDF data extractions and the corresponding JSON data generations.

## Introduction

With the increasing adoption of semantic web technologies (22–25) and formalisms in biomedical and biomolecular areas, many popular database applications (such as Uniprot (36), Ensembl (9), BioModels (19), etc.) provide accessible data represented in a Resource Description Framework (RDF) format (10, 13, 27). As the World Wide Web Consortium (W3C) recommended standard, the graph-based RDF model is well suitable for explicitly publishing life science data and linking the diverse data resources (5, 7, 11, 28). For instance, the RDF model is chosen in GlycoRDF (32) for the glycomics-based data resource integration and representation. In (31), by using the RDF model, the DisGeNET platform interconnects multiple gene-disease associations and pharmacological data sources obtained from several drug discovery applications for helping us study molecular mechanisms underpinning human diseases. To effectively publish the cross-reference information about diseases and abnormal states extracted from disease ontology and abnormality ontology, Disease Compass (17) linked the causal chains of diseases by using the RDF model.

The emergence of numerous RDF-based applications lead to the generation of massive RDF data resources, which naturally attracts the interest of seeking for novel architectures and providing supports for the future RDF data explosion (2). In recent years, JSON (JavaScript Object Notation) becomes a popular format for representing and publishing massive data resources over the web application (8, 16, 18, 35, 38). JSON documents could be used to store records in MongoDB database (3), which is a cross-platform and support distributed processing of data sets with a large size. Previous attempts (4, 14) partitions RDF data graph into several subgraphs by duplicating no-literal nodes, and then organizes these partitioning subgraphs in MongoDB. This partitioning approach duplicates no-literal nodes twice and costs some extra storage space.

JSON has been accepted as a major format for the future data explosion in National Center for Biotechnology Information dbSNP (34). Now the architecture of dbSNP is redesigned to provide products by using JSON files, which suit for the programmatic approaches well and could effectively provide supports for the increasing volume of data. Orphanet (26) chooses JSON as a new data format for the mission providing the scientific community with freely available data sets related to rare diseases and orphan drugs in a reusable format. As a completely language-independent format, JSON has a higher compression ratio in coding, and this property makes it take up less space (30). This property also makes JSON become a popular format for data interchange on the web (15, 20, 21, 39). Therefore, many web-based services, such as Semantic-JSON (16), Open chemistry (12), etc., choose JSON as the data representation formats. In order to provide access to the linked life science database, Semantic-JSON (16) develops an interface using a representational state transfer (REST) web service to the retrieval of the Semantic Web data in JSON formats. Open chemistry (12) develops a web application by providing access to the open source chemical science data in JSON formats. It facilitates data exchanges across different languages, and makes it easy to send the chemical science data to the web client developed in JavaScript. Moreover, the Ensembl (39) provides a web service to retrieves its data through the REST service, and JSON is chosen as its main endpoint data exchange format.

The adoption of a new data format will trigger the requirement of data migrations from the historical format to the new one (33). There have been some mapping tools that allow data migrations in previous works, e.g. Bio2RDF (6) and VCF2RDF (29), etc. Bio2RDF creates a knowledge space based on RDF documents by converting public bioinformatics databases into RDF documents and linking them together with normalized URIs (Uniform Resource Identifiers) in a standardized way. VCF2RDF presents the isomorphic mapping between VCF and RDF to make portability and interoperability of the self-contained description of the genomic variation in next-generation sequencing results. Unfortunately, although JSON has been employed to model future data explosion, and the available data size of RDF sources is rapidly increasing, relatively little work focuses on the data migrations from RDF to JSON (1, 37). In particular, in the new era of big data, the studies on the mapping rules and an effective mapping tool from RDF to JSON for biomedical users without programming skills obviously lag behind.

Currently, developing an effective mapping technique deal with data migrations from the RDF model to the JSON model in a uniform way is still an open problem. In order to solve this problem, in this paper we present an effective mapping tool that allows data migrations from RDF to JSON for supporting future massive data explosion. After giving a set of mapping rules which transform the RDF format into the JSON format, the corresponding transformation algorithm from RDF to JSON is presented. On this basis, we develop a user-friendly tool called RDF2JSON, which enables automating the process of the RDF data extraction and the corresponding JSON data generation. Finally, experimental evaluations are carried out to verify the advantages of the proposed tool by using real-world data sets.

## Materials and methods

In this section, we firstly give the RDF graph model parsing process, and then introduce the mapping rules. Based on these mapping rules to transform, the corresponding data migration algorithm is developed. The implementation details about the mapping tool RDF2JSON is given in the end of this section.

### RDF graph parsing

**Figure 1:**
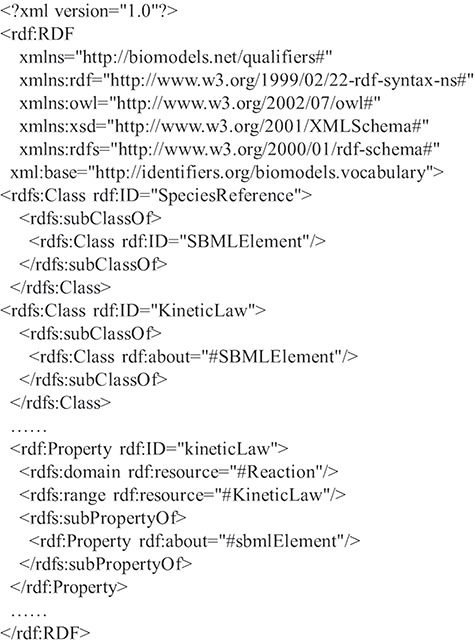
A fragment of an RDF schema.

RDF is based on the triples consisting of resources, properties and values. A resource is an entity accessible by an URI, a property defines a binary relation between resources or literals and a value is a literal or a resource. RDF Schema (RDFS) (27) provides a data modeling vocabulary and syntax to RDF descriptions. RDFS allows the definitions of the class and the property, respectively, which have global effects. It is flexible to add new properties to existing classes. In the following, a fragment of an RDF schema about computational models of biological processes is given in Figure 1.

In Figure 1, there are some classes such as `SBMLElement’, `SpeciesReference’ and `KineticLaw’, and some properties such as `sbmlElement’ and `kineticLaw’. Since classes and properties can be refined in subclasses and subproperties, there is a hierarchy of classes and a hierarchy of properties existing in RDFS. For example, the tag `rdfs:subClassOf’ represents that the class `KineticLaw’ is a subclass of the class `SBMLElement’, and the tag `rdfs:subPropertyOf’ depicts that the property `kineticLaw’ is a subproperty of the property `sbmlElement’. In the domain and range mechanisms of RDFS, a property is defined according to a domain and has an associated range that can be a literal or a class, which build the relations among classes and properties. For example, the property `kineticLaw’ is limited by the property `domain’ and the property `range’, which means that the types of values are instances of the class `KineticLaw’ and the class to which the property ascribes to is `Reaction’.

**Figure 2:**
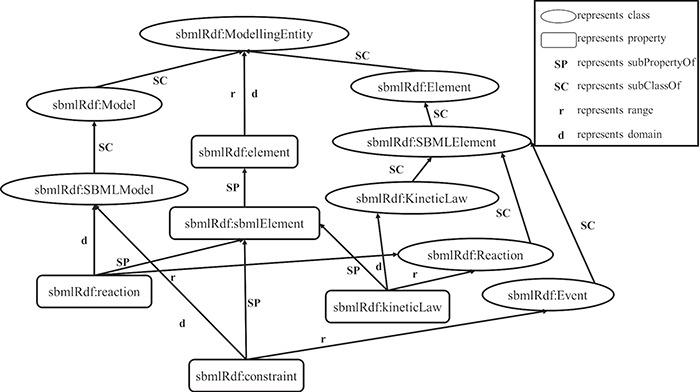
The partial graph model based on the given instance of RDFS.

An RDF statement consists of a subject, a predicate and an object, in which the subject is a class and the object is a class or a literal, the predicate is a property. Since a hierarchy of classes and a hierarchy of properties are built by RDFS using the vocabulary in the RDF statements, we can take advantage of this hierarchy to construct an RDF graph model for data extractions. Figure 2 shows the constructed graph model of the RDF schema above based on the hierarchy relationships of classes and properties. In particular, in this graph model, vertices depict the classes and literals, and edges represent properties.

### Mapping rules from RDF to JSON

This section will introduce the mapping rules from RDF to JSON. The details of the mapping rules are shown as follows.

**RULE 1** For the root element (depicted as rdf: RDF) in the RDF document, the namespaces are transformed in the following format:

“namespaces”: {prefix: namespace, …},

where the prefix is to refer to the RDF namespace, and the namespace is identified by the URI.

**RULE 2** For the basic RDF description, the key is the property. The value of the property is divided into two categories to describe:

(a) Literal type: The value object includes value and datatype. If the language property of the property value is specified, the language attribute (*lang*) is also included.
(b) URIRef type: The value object includes `rdf:resource’, which is called by all things described by RDF.

**RULE 3** For the value of the property being a blank node identifier, all the triples, in which the identifier is the resource, are extracted from the model. And these triples are processed recursively according to the basic RDF description.

In the case where the value of a property is a set of values, the W3C recommendation defines container such as bags, sequences and alternatives in order to hold such values. The parsing rules are following:

**RULE 4** For a set of values, the following elements under the child relation are mapped into key-value pairs, in which the key is the ordinal attribute and the value is the value of the element. The type of the container is retained.

For describing a group containing only the specified members and complying with the grammar rules of the triple, the elements are nested according to the order of elements.

**RULE 5** For a group of the specified elements, the nested structure is parsed to generate a list of key-value pairs and the type information is retained.

**RULE 6** For the hierarchy relation of classes, the key-value pair, in which the key is the property name of subclass and the value is the URI of superclass, is put under the resource JSON object.

**Figure 3:**
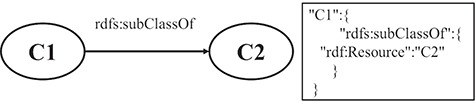
A mapping example of the hierarchy relation of classes.

Figure 3 shows a schematic diagram for the process of hierarchy relation of the class, in which C1 and C2 are class instances. The solid line with label represents that C1 is a subclass of C2. The relation is extracted from the triple set of the description about C1. The relation is transformed into a pair of key and value, which is put under the JSON object of C1.

**RULE 7** For the hierarchy relation of properties, the key-value pair, in which the key is the property name of the subproperty and the value is the URI of super property, is put under the resource JSON object.

**Figure 4:**
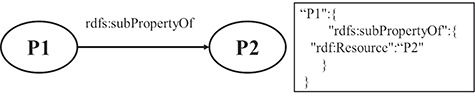
A mapping example of the hierarchy relation of properties.

Figure 4 shows a schematic diagram for the process of hierarchy relation of property. The solid line with label represents that P1 is a subproperty of P2, and P1 and P2 are property instances. The relation is extracted from the triple set of the description about P1. The relation is transformed into a pair of key and value, which is put under the JSON object of P1.

**RULE 8** For the domain and range of a property, two key-value pairs, in which the keys are the names of properties and the values are the value of the property, are put under the property JSON object.

**Figure 5:**
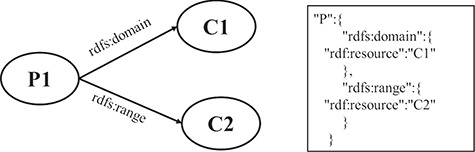
A mapping example of the domain and the range of properties.

Figure 5 shows a schematic diagram for the process of the domain and range of a property. C1 and C2 are class instances defining the resources denoted by the subjects and objects of the triples whose predicate is P. The relation is extracted from the triple set of the description about P. The relations are transformed into two pairs of key and value, which are put under the JSON object of P.

**RULE 9** For repetitive properties of the same resource, the values are merged into an array.

**RULE 10** For the property that does not contain a specific value, a blank string is used to describe the value.

**Figure 6:**
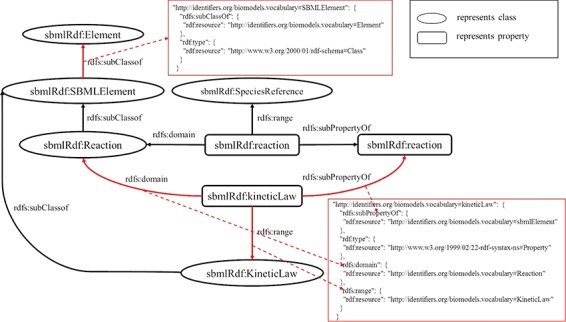
A mapping instance from RDF to JSON.

Figure 6 shows an example that illustrates the mapping between RDF and JSON. For simplicity, only two representative instances are shown in the figure. The property `sbmlRdf:kineticLaw’ is defined with domain property `sbmlRdf:Reaction’ and range property `sbmlRdf:KineticLaw' and has a superproperty *`*sbmlRdf:sbmlElement’. According to rules 7 and 8, these properties could be mapped into the JSON object of the property `sbmlRdf:kineticLaw’, which has been defined by the base descriptions of the property `sbmlRdf:kineticLaw’ according to rules 2 and 3. Similarly, the class `sbmlRdf:SBMLElement’ has a superclass `sbmlRdf:Element’, and the hierarchical relationship could be mapped into the JSON object of the class `sbmlRdf:SBMLElement’ according to rule 6.

**Figure 7:**
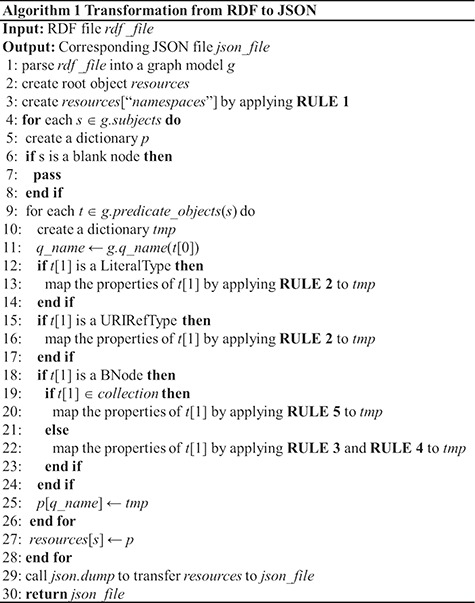
The mapping algorithm.

Figure 7 gives the data migration process based on the above mapping rules (Algorithm 1). First, we use RDFLib to parse the RDF file into a graph model. RDFLib is a python library for working with RDF, a simple yet powerful language for representing information. Then, the namespaces used to abbreviate the URI are extracted from the model into the root object by the rule1. In the main loop (4–28), all the resources contained in the model are processed step by step. And the properties associated with each resource are extracted to form the next layer of the loop (9–28). In the inner loop, we map the properties according to rules 2 to 7. Finally, the root object is mapped to the JSON file. Since the algorithm mainly consists of two layers of loop, the time complexity is O(n^2^).

### RDF2JSON framework and implementation

**Figure 8:**
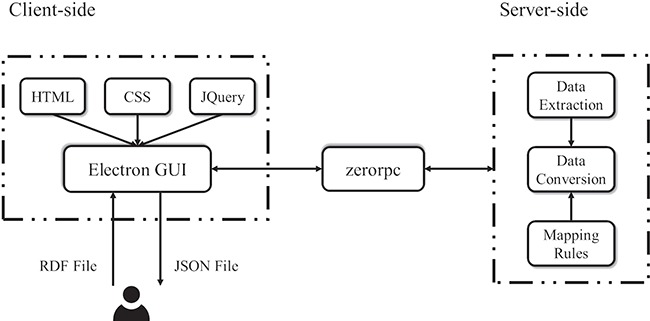
The framework of RDF2JSON.

Figure 8 shows that the RDF2JSON application is designed and implemented on the basis of client–server architecture with the intention of utilizing and separating each module into independent pieces. The server-side component is implemented by Python (version 3.6) providing access to using several libraries such as zerorpc and rdflib. Zerorpc (version 0.9.7) is a flexible remote procedure call implementation, which serves as a known remote server to execute a specified procedure with supplied parameters. The pre-processing of the data conversion uses the rdflib library to parse the input file to get RDF statements before applying the mapping rules. To write the transformed data into the output file we use JSON module in Python. The returned data is saved under the same path of the source.

The client-side component handles interaction with user, which is mainly implemented in electron (version 6.0.0), an open-source framework for creating native applications with web technologies such as JavaScript, HTML5 and CSS. The page architecture, design and functionality use the Bootstrap framework together with several jQuery (version 3.x) plugin to enhance user interactivity. To improve user friendliness, a common display layout is adopted and maintained between application functions. The file upload area is located on the right side of the page. As a desktop application, it allows a cross-platform compatibility among the most used operating systems (Windows 7 or above) and Linux (version 16.04).

### Load JSON file into MongoDB

MongoDB could use the transformed JSON files to store massive records obtained from RDF files. The mongoimport tool is provided for importing the JSON file into MongoDB; the system command line is as follows:

mongoimport –d < database> --c <collection> --file <filename>,where “--d” specifies the name of the database, “--c” specifies the collection to import and “--file” specifies the location and name of a file containing the data to import. Note that the field names of the document cannot contain “.” or null, and cannot start with “$” for the system reference. Thus, if these characters appear in the document fields, they need to be replaced with other specific character such as Unicode characters.

After storing the RDF records in MongoDB, the basic MongoDB query mechanism could be used for searching the desired results. In particular, a `find’ function, which receives two parameters in JSON documents, `query’ and `projection’, and returns a cursor to the matching documents, could be used for the searches. The goal of `query’ is to specify the conditions that determine which documents to select, in which the <field>:<value> expressions is used to specify the equality conditions and query operator expressions. The `projection’ is used to specify the fields to return in the documents. The MongoDB query also provides aggregation pipeline methods called `aggregate’ to process a series of documents (such as COUNT, GROUP, SORT, LIMIT, SKIP, etc.) to provide aggregate computations. Besides, the expression operators can be used to construct the query expressions such as arithmetic expression operators, boolean expression operators, etc.

## Results

In order to test the performance of RDF2JSON, two real-world data sets UniprotKB and BioModels containing RDF data sources are chosen in our experiments. UniProtKB is a comprehensive resource for protein sequence and annotation data. BioModels is a repository of computational models of biological processes. All the experiments are running on the Ubuntu 18.04 LTS 64-bit operating platform with the following system features: Intel® core i5-8500 CPU @3.00GHz×6 and 32GB main memory. The approaches are programmed in Python 3.6.

Table 1 gives the results of the experiment running on the UniprotKB data set. From Table 1, we observe that, for the experimental data set, JSON provides a compression storage and the compression rate for the tested data is about 40%. The best compression happens in R1 with the rate of 46% (the size of using RDF is 8.75 MB and the size of using JSON is 4.64 MB). As the RDF is an extension of XML and it is a complete markup language, it uses redundant tags for the content descriptions, which may result in redundant storage. JSON organizes data as an ordered list of `name/value’ pairs, which reduces the redundant tags.

**Table 1:** Experimental results running on UniprotKB

	File name	The numbers of subjects	RDF size (MB)	JSON size (MB)
R1	uniprotkb_eukaryota_oxymonadida_66288.rdf	11 662	8.75	4.64
R2	uniprotkb_eukaryota_glaucocystophyceae_38254.rdf	35 767	23.41	13.71
R3	uniprotkb_eukaryota_alveolata_33630_1000000.rdf	763 169	541.61	310.40
R4	uniprotkb_eukaryota_rhizaria_543769.rdf	1 973 509	1307.53	785.36
R5	uniprotkb_eukaryota_parabasalia_5719.rdf	2 424 903	1571.67	950.61
R6	uniprotkb_eukaryota_rhodophyta_2763.rdf	4 031 384	2678.03	1583.91
R7	uniprotkb_eukaryota_opisthokonta_fungi_4751_8000000.rdf	4 448 979	3173.19	1815.40

**Figure 9:**
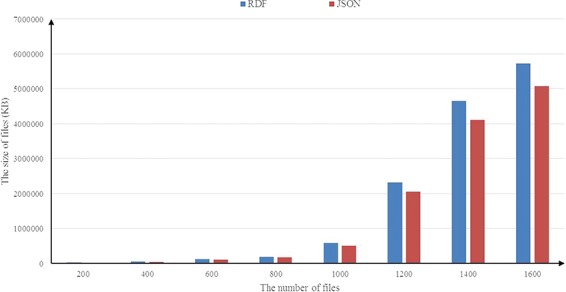
Occupied storage comparisons.

**Figure 10:**
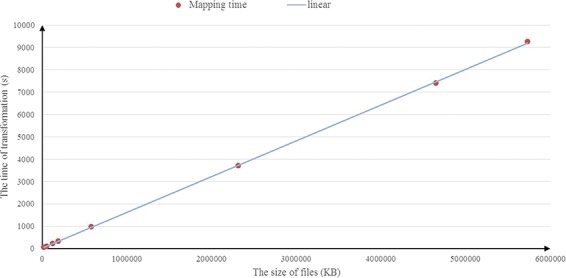
Running times by varying the input size.

Figure 9 illustrates the results of the experiments running on the BioModels data set. In Figure 9, similar to the experimental results obtained in the UniprotKB data set above, a consistent result is observed and the average compression rate is about 14%. We also investigate the scalability of RDF2JSON, by varying number of the input RDF files. Figure 10 reports the running times when the size of input RDF files increase. From the figure, we can see that the running time will increase when the input files increase, and it approximates linear growth.

## Discussion

With the emergence of rapidly increasing size of RDF data sources, there is an urgent need of a novel infrastructure to provide effective supports for future data explosion. In this paper, we deal with the RDF data explosion issue by introducing the JSON model, and show the benefits of using JSON. After parsing the RDF schema, we present a set of mapping rules, which transforms an RDF schema into the JSON schema, and then propose an effective and specified algorithm to complete data migrations from RDF to JSON. We complement the work with a user-friendly and cross-platform tool RDF2JSON to help users without programming skills. Through the final experiment results, we also demonstrate the performance and advantages of RDF2JSON by using the real-world Uniprot and BioModels data sets. In the future work, we plan to integrate efficient query processing and optimization approach by using Spark/MapReduce, which has promising processing performance over massive data.

## Notes

**Database URL:** https://github.com/lyotvincent/rdf2json
